# SNRPB promotes the progression of hepatocellular carcinoma via regulating cell cycle, oxidative stress, and ferroptosis

**DOI:** 10.18632/aging.205371

**Published:** 2024-01-05

**Authors:** Xiaoyan Wang, Hao Zhang, Zehao Guo, Junyuan Wang, Chuntao Lu, Junhua Wang, Rongzhong Jin, Zhijing Mo

**Affiliations:** 1 Department of Experimental Teaching Center, School of Intelligent Medicine and Biotechnology, Guilin Medical University, Guilin 541199, Guangxi, China; 2 Key Laboratory of Biochemistry and Molecular Biology, Guilin Medical University, Education Department of Guangxi Zhuang Autonomous Region, Guilin 541199, Guangxi, China; 3 Department of Biomedical Engineering, School of Intelligent Medicine and Biotechnology, Guilin Medical University, Guilin 541199, Guangxi, China; 4 Department of Biochemistry, School of Intelligent Medicine and Biotechnology, Guilin Medical University, Guilin 541199, Guangxi, China

**Keywords:** SNRPB, HCC, oxidative stress, cell cycle, sorafenib

## Abstract

Small Nuclear Ribonucleoprotein Polypeptides B and B1 (SNRPB) have been linked to multiple human cancers. However, the mechanism of SNRPB in hepatocellular carcinoma (HCC) and whether SNRPB has a synergistic effect with sorafenib in the treatment of HCC remain unclear. In this study, bioinformatic analysis found that SNRPB was an independent prognostic factor for HCC that exerted a critical effect on the progression of HCC. SNRPB was linked with immune checkpoints, cell cycle, oxidative stress and ferroptosis in HCC. Single cell sequencing analysis found that HCC cell subset with high expression of SNRPB, accounted for a higher proportion in HCC cells with higher stages, had higher expression levels of the genes which promote cell cycle, inhibit oxidative stress and ferroptosis, and had higher cell cycle score, lower oxidative stress score and ferroptosis score. Single-sample gene set enrichment analysis (ssGSEA) analysis found that 17 oxidative stress pathways and 68 oxidative stress-ferroptosis related genes were significantly correlated with SNRPB risk scores. SNRPB knockdown induced cell cycle G2/M arrest and restrained cell proliferation, while downregulated the expression of CDK1, CDK4, and CyclinB1. The combined treatment (SNRPB knockdown+sorafenib) significantly inhibited tumor growth. In addition, the expression of SLC7A11, which is closely-related to ferroptosis, decreased significantly *in vitro* and *in vivo*. Therefore, SNRPB may promote HCC progression by regulating immune checkpoints, cell cycle, oxidative stress and ferroptosis, while its downregulation inhibits cell proliferation, which enhances the therapeutic effect of sorafenib, providing a novel basis for the development of HCC therapies.

## INTRODUCTION

The sixth most common cancer worldwide is primary liver cancer, which is also the third most lethal cancer [1]. In 2020, 905,677 new cases and 830,180 deaths were reported [1]. In China, more than 41 thousand new cases along with 39 thousand deaths were reported [2], while HCC accounted for 75% to 85% of primary liver cancer cases [1]. For this reason, it is very important to identify target molecules associated with the genesis and development of HCC and study their molecular mechanisms for the prevention and treatment of HCC.

The key component of the spliceosomal U1, U2, U4, and U5 (snRNP) is SNRPB, which plays a pivotal role in cell pre-mRNA splicing [3]. SNRPB has been linked to various human cancers. For example, SNRPB is related to lung cancer [4], its knockdown inhibits the cell proliferation of glioblastoma cells [5], it accelerates the progression by regulating Rab26 [6] and negatively regulates the resistance of cis-platinum in non-small cell lung cancer cells [7], and it accelerates the progression of cervical cancer through the inhibition of p53 expression, which has an impact on cell cycle [8]. Recent research has shown that SNRPB plays a vital role in HCC as well [9, 10]. For example, C-MyC mediates SNRPB upregulation and induces the proliferation and migration of HCC cells [9], while SNRPB over-expression accelerates the malignant proliferation and stemness maintenance of these cells [10]. However, it remains unclear whether SNRPB has an influence on the HCC cells proliferation through cell cycle regulation or cell death.

Ferroptosis is a kind of cell death induced by accumulated iron-dependent lipid reactive oxygen species (ROS) [11, 12]. The metabolism of cancer cells is very active and has higher levels of ROS, thus are more susceptible to ferroptosis [12]. The regulatory mechanism of ferroptosis mainly involves iron transport, amino acid metabolism and lipid peroxidation [12]. Therefore, all molecules that can regulate cellular redox homeostasis, cell metabolism and iron homeostasis may affect ferroptosis. Oxidative stress results from excessive accumulation of reactive oxygen produced by aerobic metabolism of organisms which may lead to cell death [13]. Therefore, whether SNRPB is related to oxidative stress and ferroptosis in HCC is worthy of further investigation.

The influence of SNRPB on HCC progression was analyzed using bioinformatics and found that SNRPB is related to cell cycle, oxidative stress and ferroptosis in HCC. Single cell sequencing analysis showed that high expression of SNRPB can promote cell cycle progression of HCC cells, while downregulation of SNRPB can promote oxidative stress and ferroptosis of HCC cells. Our experiments revealed that knockdown SNRPB inhibits the expressions of CDK4 and CDK1/CyclinB1 and induces G2/M phase arrest. At the same time, it also inhibits SCL7A11 protein expression, and promotes ferroptosis in HCC cells, while the combination of SNRPB and sorafenib have a better efficacy. Collectively, SNRPB exerts a critical effect on promoting the progression of HCC.

## RESULTS

### SNRPB and clinicopathological features and survival rates of HCC patients

**Figure 1:**
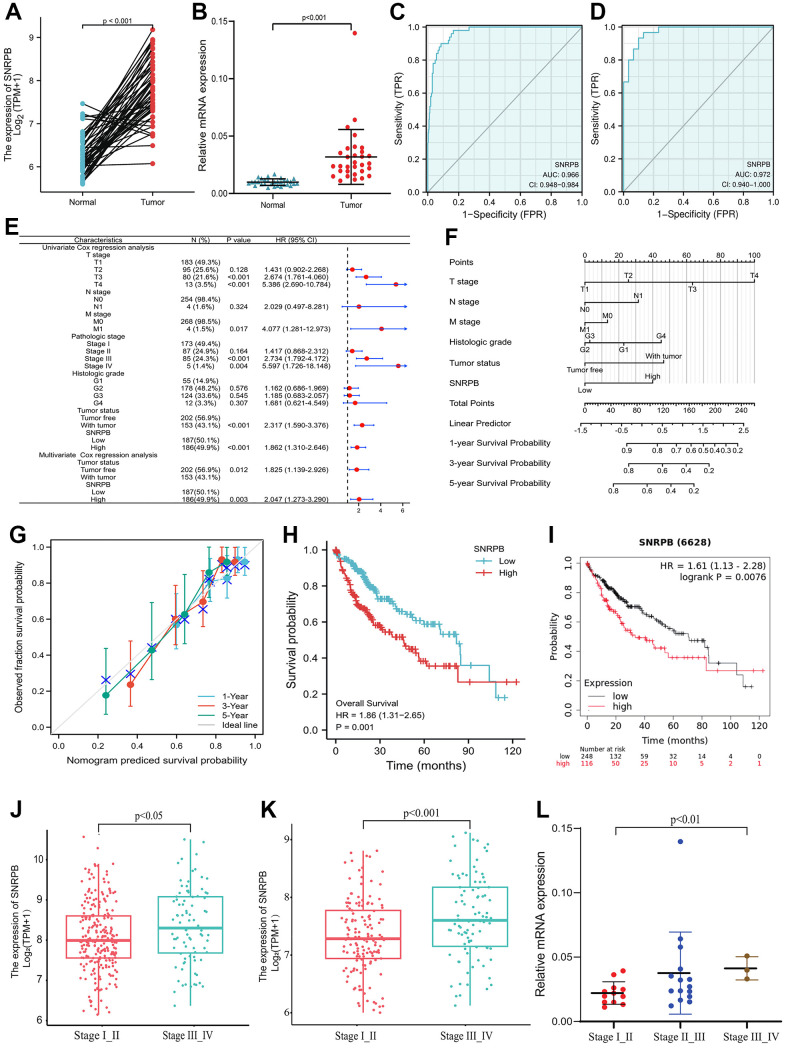
**Correlation of SNRPB and the clinicopathological features and survival rates of patients with HCC.** (**A**) mRNA relative expression levels of SNRPB in HCC tissues and paired adjacent tissues acquired from TCGA. (**B**) mRNA relative expression levels of SNRPB in 30 HCC tissues and 30 paired adjacent tissues. (**C**) ROC analysis of the diagnosis of SNRPB in HCC patients using data obtained from TCGA. (**D**) ROC analysis of SNRPB expression in 30 HCC and 30 paired adjacent tissues. (**E**) Forest plot using univariate and multivariate Cox regression analysis. (**F**) Nomogram of the overall survival (OS) for patients with HCC (1, 3, and 5 years). (**G**) Calibration curve of OS at 1, 3 and 5 years. The gray diagonal is the ideal case line and the blue cross represents the result of hierarchical Kaplan-Meier correction for each point. (**H**) Relationship between OS and SNRPB expression in TCGA. (**I**) Relationship between OS and SNRPB expression obtained using the Kaplan-Meier Plotter. (**J**) Relationship between HCC stages and the expression level of SNRPB in TCGA dataset. (**K**) Relationship between HCC stages and the expression level of SNRPB in ICGC dataset. (**L**) Relationship between HCC stages and the expression level of SNRPB in 30 HCC tissues.

As shown in Figure 1A, the paired samples showed that SNRPB expression was upregulated in HCC tissues with statistical significance compared to adjacent normal tissue (*p* < 0.001). SNRPB expression can be used to distinguish cancer tissues from the adjacent tissues with a high level of credibility (Area Under Curve, AUC = 0.966) (Figure 1C). Then, RT-qPCR analysis conducted on 30 paired HCC and normal tissue samples confirmed that SNRPB was notably upregulated in HCC tissues (Figure 1B), and that it could distinguish cancer tissues from adjacent normal tissues with a high level of credibility (AUC = 0.972) (Figure 1D). SNRPB and T stage, M stage, pathological stage, tumor status and other characteristics were positive correlation with statistical significance (Figure 1E). The nomogram showed that a higher level of SNRPB expression had a larger contribution to the prediction model, and a lower survival rate at 1, 3, and 5 years in HCC patients (Figure 1F). The calibration curve confirmed the accuracy of the prediction model because actual survival at 1, 3 and 5 years was shown to be in line with the ideal gray line (Figure 1G). Additionally, Cox regression analysis indicated the significant correlation between the high SNRPB expression and the poor prognosis of HCC patients (Figure 1H), which was confirmed by the results of 364 samples from the Kaplan-Meier Plotter database (Figure 1I). The HCC stages from The Cancer Genome Atlas (TCGA) (*p* < 0.05) (Figure 1J) and International Cancer Genome Consortium (ICGC) database (*p* < 0.001) (Figure 1K) were positive correlation with SNRPB, which was confirmed by RT-qPCR analysis of 30 HCC tissue samples (*p* < 0.01) (Figure 1L). Higher SNRPB expression may be an influencing factor of poor prognosis and is positively associated with the progression of HCC.

### SNRPB expression and immune cell infiltration in HCC

**Figure 2:**
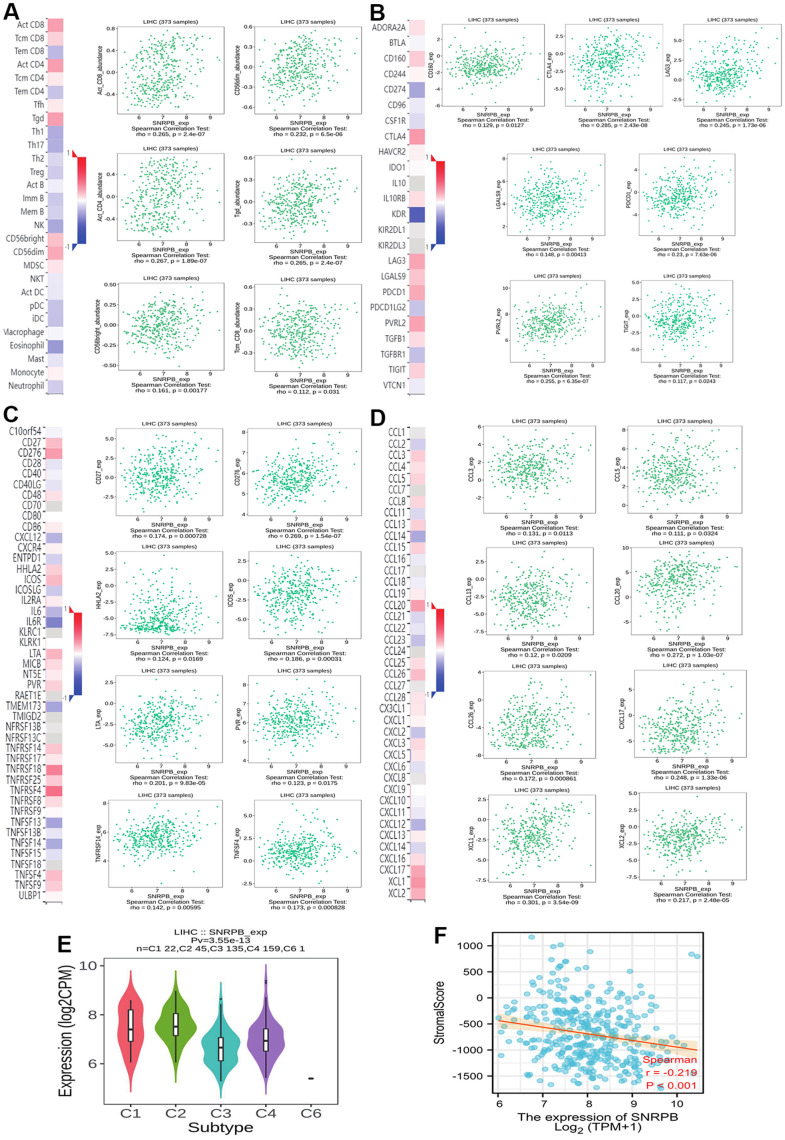
**Correlation between SNRPB and immune cell infiltration in HCC.** (**A**) Correlation between SNRPB expression and immunity cells. (**B**) Correlation between SNRPB expression and checkpoints. (**C**) Correlation between SNRPB expression and immune inhibitors. (**D**) Correlation between SNRPB expression and chemokines. (**E**) SNRPB expression in different HCC immune subtypes. (C1 (wound healing), C2 (IFN-gamma dominant), C3 (inflammatory), and C4 (lymphocyte depleted)). (**F**) Correlation between SNRPB expression and immune stroma score.

To explore the relationship of SNRPB with immunity, we first compared the distribution of immune cells under different SNRPB expression levels via TISIDB. We discovered that the distribution of six immunity cells, including activated CD8^+^ T (Act CD8) cells, central memory CD8^+^ T (Tcm CD8) cells, and gamma-delta T (Tgd) cells were positively related to SNRPB expression, and the distribution of thirteen immunity cells, including effector memory CD8+ T (Tem CD8) cells, T helper 1 (Th1) cells, and plasmacytoid dendritic cells (pDC) were negatively related to SNRPB expression (Figure 2A). In addition, seven key immune checkpoints, including the CD160, CTLA4, LAG3, LGALS9, PDCD-1, CD112 (PVRL2), and TIGIT (Figure 2B), eleven key immune inhibitors, including CD27, CD276, and HHLA2 (Figure 2C), and eleven chemokines, including CCL3, CCL5, and CCL8 (Figure 2D) were significantly positively related to SNRPB expression. SNRPB expression was significantly differences among the different HCC immune subtypes and was higher in C2 (C2 > C1 > C4 > C3) (Figure 2E). SNRPB expression was also negatively related to the immune stroma score (Figure 2F).

### Single cell RNA sequencing analysis of SNRPB

**Figure 3:**
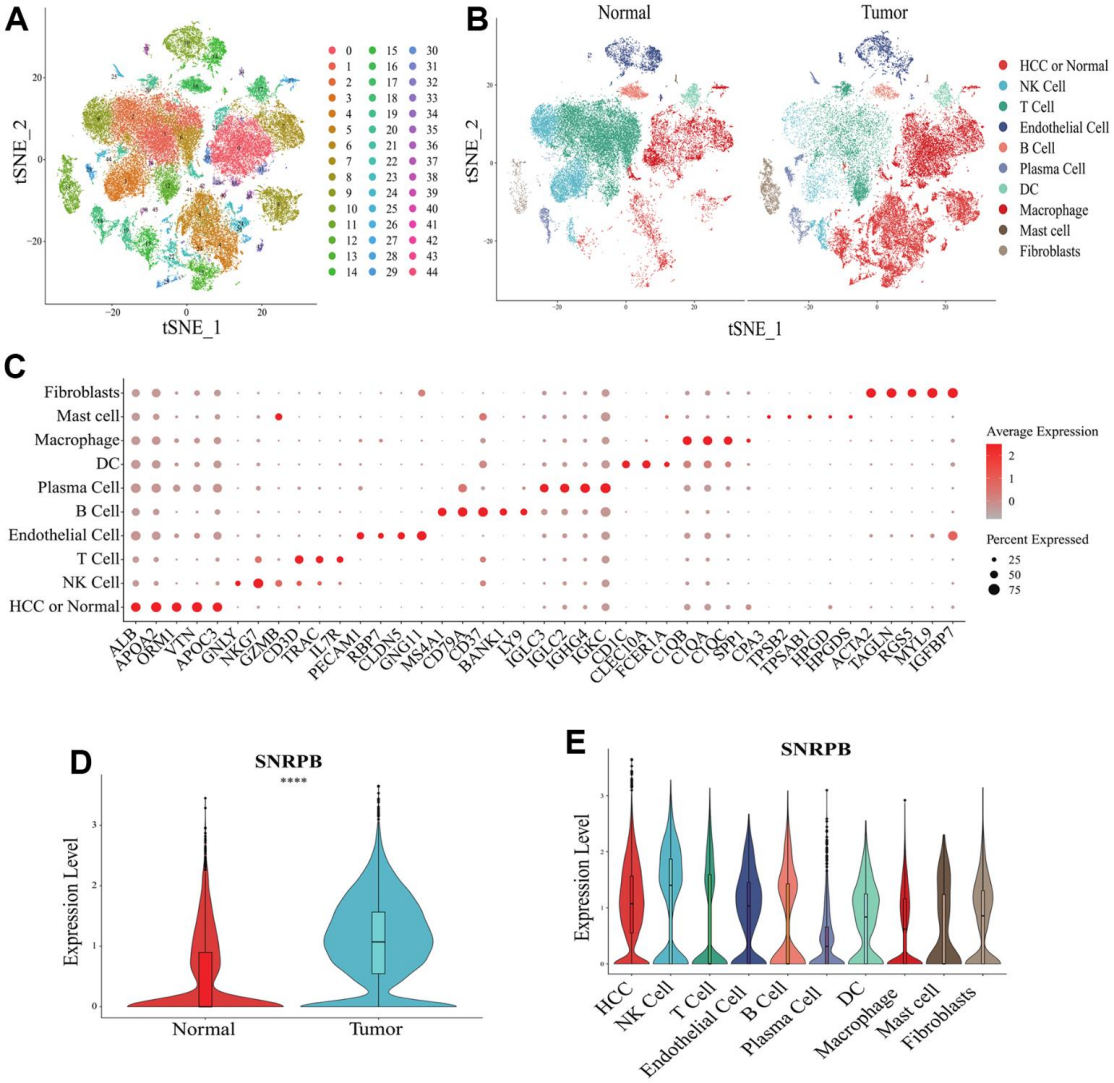
**Single cell RNA sequencing analysis of SNRPB.** (**A**, **B**) t-SNE dimensionality reduction. (**C**) Bubble plot showing the expression levels of cluster-specific marker genes. The size of the dots represents the percent of expressed cells, and the color of the dots represents the average expression levels. (**D**) The expression levels of SNRPB in HCC cells and normal epithelial cells. (**E**) The expression levels of SNRPB in 10 cell types.

**Figure 4:**
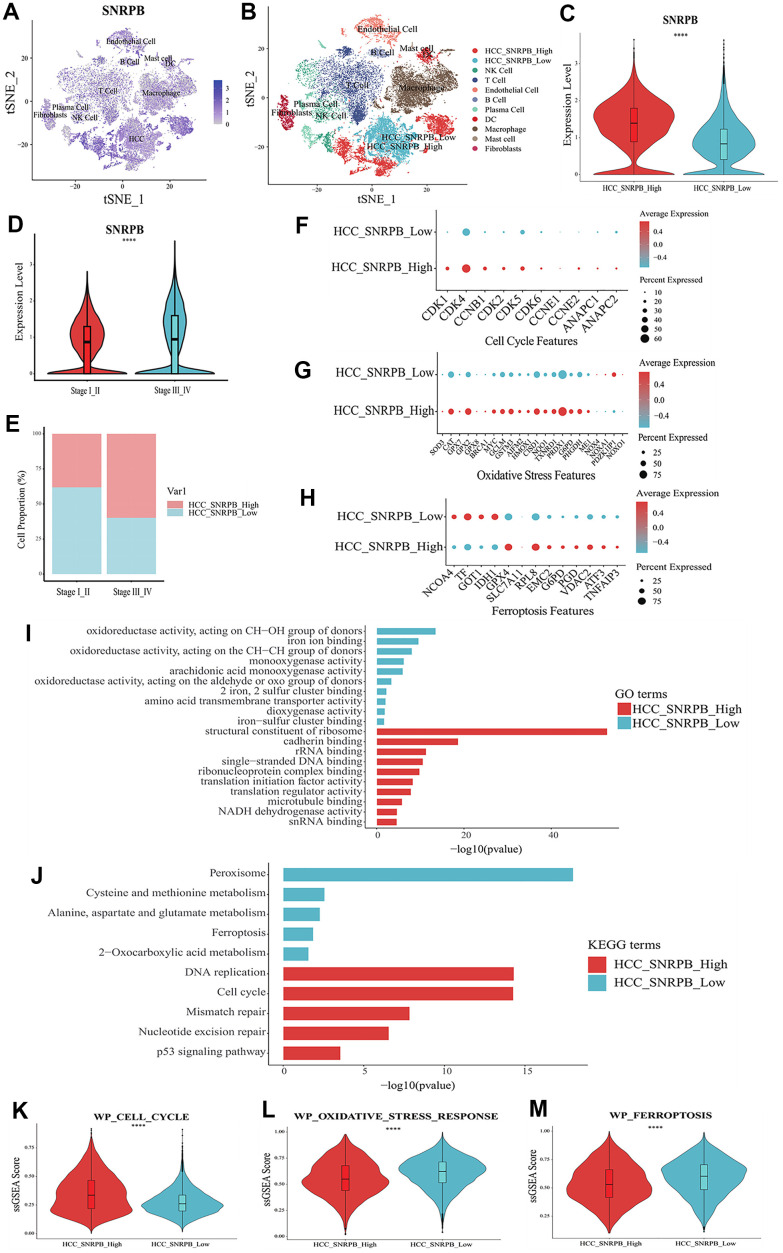
**Single cell RNA sequencing analysis of the relationship between SNRPB and cell cycle, oxidative stress and ferroptosis in HCC.** (**A**) t-SNE dimensionality reduction of the SNRPB expression levels in HCC samples. (**B**) HCC cells were divided into high expression of SNRPB subset (HCC_SNRPB_High) and low expression of SNRPB subset (HCC_SNRPB_Low). (**C**) Analysis of SNRPB expression between HCC_SNRPB_High and HCC_SNRPB_Low groups. (**D**, **E**) Relationship between HCC stages and the expression level of SNRPB in HCC_SNRPB_High and HCC_SNRPB_Low groups. (**F**–**H**) The expression levels of cell cycle, oxidative stress, and ferroptosis-related genes in HCC_SNRPB_High and HCC_SNRPB_Low groups. The size of the dots represents the percent of expressed cells, and the color from red to blue represents the average expression level from high to low. (**I**) GO enrichment analysis of HCC_SNRPB_High and HCC_SNRPB_Low groups. (**J**) KEGG enrichment analysis of HCC_SNRPB_High and HCC_SNRPB_Low groups. (**K**–**M**) ssGSEA scores of cell cycle, oxidative stress, and ferroptosis-related pathways in HCC_SNRPB_High and HCC_SNRPB_Low groups.

t-distributes stochastic neighbor embedding (t-SNE) analysis showed that a total of 44 cell clusters were obtained from 10 HCC samples and 8 normal samples (Figure 3A). The cell clusters were annotated according to cluster-specific marker genes, and the 44 cell clusters were divided into 10 cell types, including HCC or normal epithelial cells, natural killer (NK) cells and plasma cells (Figure 3B). The specific marker genes expression of 10 cell types were visualized (Figure 3C). Compared with normal epithelial cells, SNRPB was significantly higher expressed in HCC cells (Figure 3D), and SNRPB was expressed in all 10 cell types, with the highest expression level in NK cells and the lowest expression level in plasma cells (Figure 3E).

t-SNE analysis showed that SNRPB was expressed in all 10 cell types (Figure 4A). HCC cells were divided into two subsets, HCC cells with high expression of SNRPB (HCC_SNRPB_High) and HCC cells with low expression of SNRPB (HCC_SNRPB_Low) (Figure 4B). The SNRPB expression in HCC_SNRPB_High cell subset was significantly higher than that in HCC_SNRPB_Low cell subset (*p* < 0.001) (Figure 4C). SNRPB expression in HCC cells at stage III-IV was significantly higher than that in HCC cells at stage I-II (*p* < 0.001), which was consistent with our previous results (Figure 4D). In addition, the proportion of HCC_SNRPB_High cell subset in stage III-IV HCC cells was higher than that in stage I-II HCC cells (Figure 4E). These results show that SNRPB expression is positively correlated with HCC stages. The differential expression analysis of HCC_SNRPB_High and HCC_SNRPB_Low cell subsets indicated that the expression levels of CDK1, CDK4, CCNB1, CDK2, CDK5, CDK6, CCNE1, ANAPC1 and ANAPC2, which promote cell cycle, in HCC_SNRPB_High cell subset were all higher than those in HCC_SNRPB_Low cell subset (Figure 4F). These findings indicate that the high expression of SNRPB promotes HCC progression may be related to cell cycle. The expression levels of NOX4, NOXA1, NOXO1 and PDZKIP1, which promote oxidative stress, were lower in HCC_SNRPB_High cell subset than in HCC_SNRPB_Low cell subset. The expression levels of SOD3, CAT, GPX7, GPX8, BRCA1, MYC, GCLM, GSTM3, AIFM2, HMOX1, CISD1, NQO1, TXNRD1, PRDX1, G6PD, PHGDH and ME1, which inhibit oxidative stress, were higher in HCC_SNRPB_High cell subset than in HCC_SNRPB_Low cell subset (Figure 4G). These findings indicate that SNRPB affects the progression of HCC may be related to oxidative stress. The expression levels of NCOA4, TF and IDH1, which promote ferroptosis, were lower in HCC_SNRPB_High cell subset than in HCC_SNRPB_Low cell subset. The expression levels of GPX4, SLCA11, RPL8, EMC2, G6PD, PGD, VDAC2, ATF3, and TNFAIP3, which inhibit ferroptosis, were higher than those in HCC_SNRPB_Low cell subset (Figure 4H). These findings indicate that SNRPB affects the progression of HCC and may also be related to ferroptosis.

Gene Ontology (GO) enrichment analysis showed that HCC_SNRPB_Low cell subset was mainly related to oxidative stress-related and ferroptosis-related functions (oxidoreductase activity, monooxygenase activity, iron ion binding, etc.). The HCC_SNRPB_High cell subset was mainly related to cell cycle (single-stranded DNA binding, structural constituent of ribosome, cadherin binding, etc.) (Figure 4I). Kyoto Encyclopedia of Genes and Genomes (KEGG) pathway analysis showed that HCC_SNRPB_Low cell subset was mainly related to oxidative stress-related pathways (peroxisome and 2-oxocarboxylic acid metabolism) and ferroptosis-related pathways (ferroptosis, cysteine and methionine metabolism, alanine-aspartate, and glutamate metabolism). HCC_SNRPB_High cell subset was mainly related to cell cycle-related pathways (cell cycle, DNA replication, mismatch repair, etc.) (Figure 4J). These findings indicate that downregulation of SNRPB may activate oxidative stress and ferroptosis in HCC, while upregulation of SNRPB may promote cell cycle progression in HCC.

ssGSEA analysis showed that compared with HCC_SNRPB_High cell subset, HCC_SNRPB_Low cell subset had higher ferroptosis and oxidative stress scores and lower cell cycle scores (*p* < 0.001) (Figure 4K–4M), which further indicate that downregulation of SNRPB may promote HCC cell death by inducing oxidative stress and ferroptosis, while upregulation of SNRPB may promote HCC progression by affecting cell cycle.

### SNRPB knockdown arrests cell cycle and inhibits HCC cell proliferation

**Figure 5:**
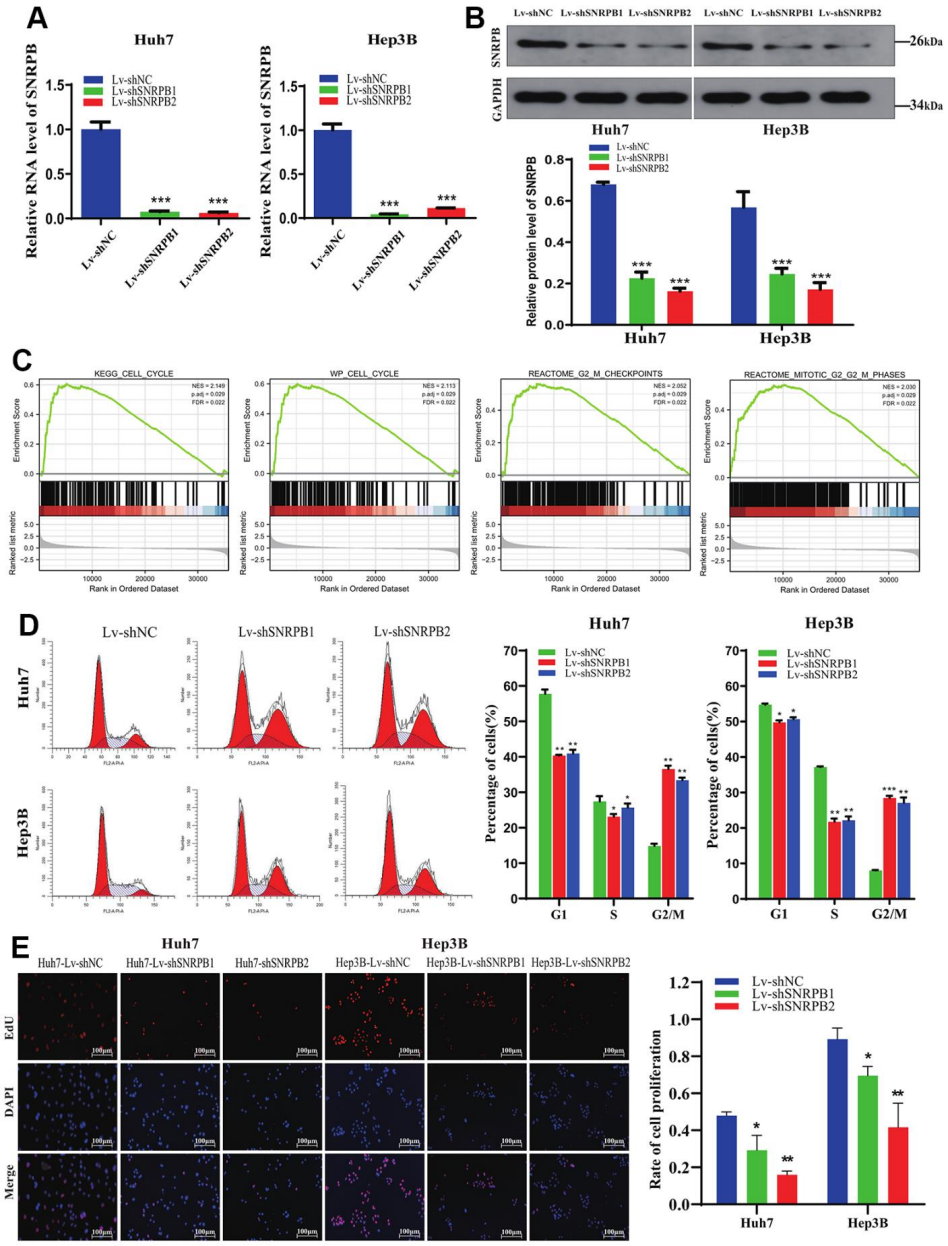
**Cell cycle arrest and proliferation inhibition of HCC cells after SNRPB knockdown.** (**A**) The mRNA expression level of SNRPB was evaluated by RT-qPCR. (**B**) The protein expression level of SNRPB was evaluated by Western blotting. (**C**) GSEA of the TCGA dataset: the SNRPB-related DGEs are enriched in the cell cycle, as well as G2/M phase. (**D**) The influence of SNRPB knockdown on cell cycle of Huh7 and Hep3B cells. (**E**) The influence of SNRPB knockdown on the proliferation of Huh7 and Hep3B cells.

To explore SNRPB function in HCC, Huh7 and Hep3B cell lines were adopted to establish SNRPB knockdown models (Figure 5A, 5B). Gene Set Enrichment Analysis (GSEA) performed on TCGA dataset revealed that the KEGG cell cycle (N = 2.113, *p* = 0.029), WP cell cycle (N = 2.149, *p* = 0.029), reactome G2/M checkpoints (N = 2.05, *p* = 0.001), reactome mitotic G2/M phases (N = 2.03, *p* = 0.001) are significantly enriched in the high SNRPB expression samples (Figure 5C). Using flow cytometry, we found a higher proportion of cells in the G2/M phase in the SNRPB knockdown group (Lv-shSNRPB1 and Lv-shSNARPB2 groups) compared with the Lv-shNC group (Figure 5D). The results of the Edu experiment demonstrated significantly lower cellular proliferation rate in SNRPB knockdown group (Lv-shSNRPB1 and Lv-shSNRPB2 groups) compared with the Lv-shNC group (Figure 5E).

### SNRPB knockdown downregulates cell cycle, oxidative stress and ferroptosis related genes

**Figure 6:**
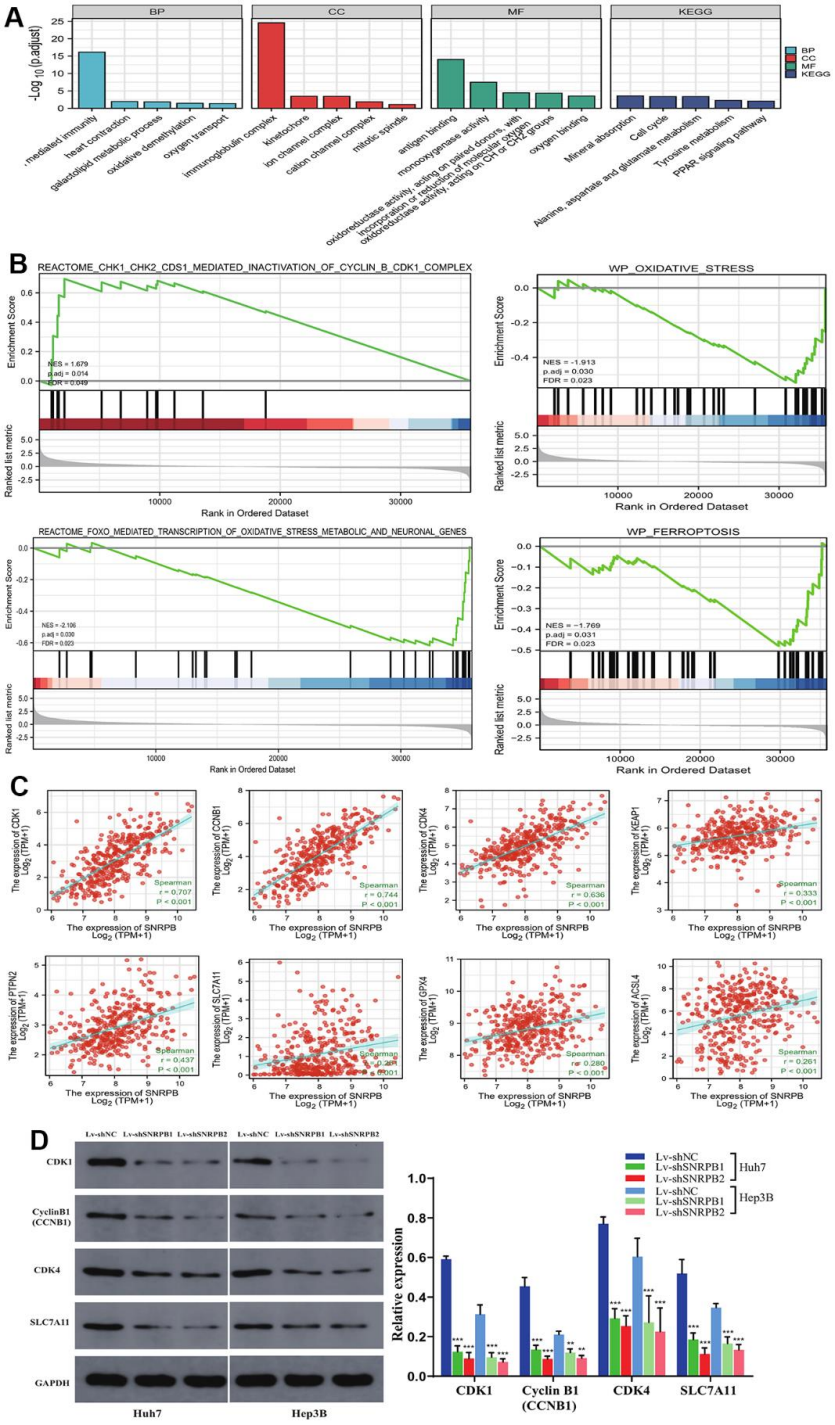
**SNRPB knockdown affects the expression of downstream genes associated with cell cycle, oxidative stress and ferroptosis.** (**A**) GO-KEGG of DEGs. (**B**) GSEA of the TCGA dataset: the high SNRPB expression associated DGEs are enriched in cell cycle related genes and that low SNRPB expression associated DGEs are enriched in oxidative stress and ferroptosis related genes. (**C**) Correlation between SNRPB expression and CDK1, CyclinB1 (CCNB1), CDK4, KEAP1, PTPN2 (TCPTP), SLC7A11, GPX4 and ACSL4 expression. (**D**) The protein expression levels of CDK1, CyclinB1 (CCNB1), CDK4, and SLC7A11 after SNRPB knockdown were evaluated by Western blotting.

SNRPB-related differentially expressed genes (DEGs) were mainly associated with oxidative stress (oxidative demethylation, oxygen transport, oxygen binding), ferroptosis (iron ion binding, glutamate metabolism), cell cycle (kinetochore, mitotic spindle, cell cycle), and cellular immunity (B cell mediated immunity, immunoglobulin complex) (Figure 6A). High SNRPB expression activated G2/M checkpoint related genes, such as cyclin B (CCNB1) and CDK1 (N = 1.679, *p* = 0.014), while low SNRPB expression activated oxidative stress genes (N = -1.913, *p* = 0.030 and N = -2.106, *p* = 0.030) and ferroptosis genes (N = -1.769, *p* = 0.031) (Figure 6B). The analysis of relationships between SNRPB expression and other related genes revealed that SNRPB was significantly positively associated with the expression of cell cycle related genes (CDK1, CDK4, CyclinB1 CCNB1), oxidative stress-related genes (KEAP1, PTPN2 (TCPTP), NOX4) and ferroptosis-related genes (SLC7A11, GPX4, ACSL4) (Figure 6C). Western blotting further confirmed that the expression of CDK4, CDK1, CyclinB1, and SCL7A11 was downregulated after SNRPB downregulation, which is consistent with previous bioinformatics analysis results. These results indicate that SNRPB downregulation may induce G2/M phase arrest, promote ferroptosis, and inhibit cell proliferation (Figure 6D).

**Figure 7:**
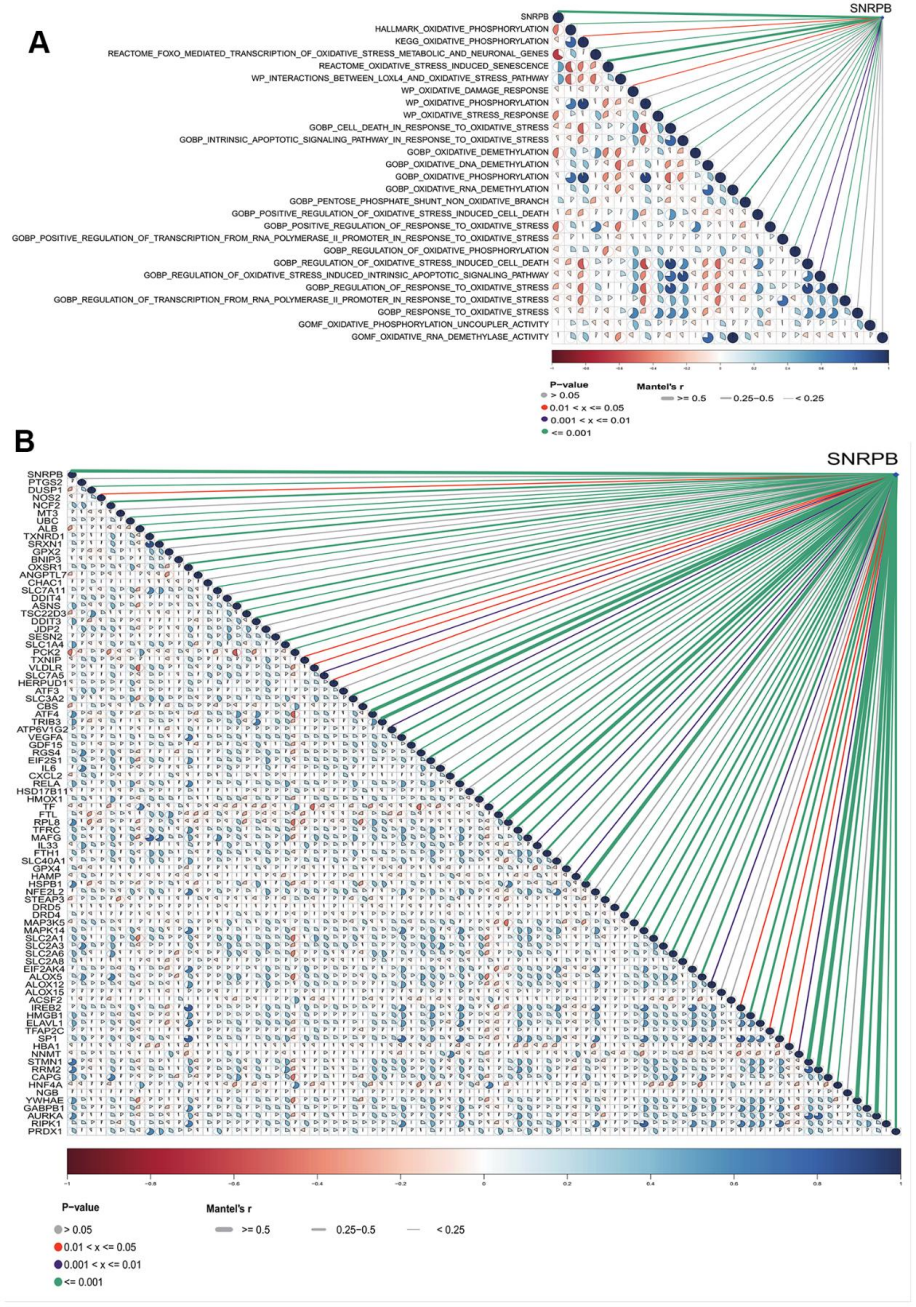
**Relationship between SNRPB and oxidative stress-related pathways and oxidative stress-ferroptosis related genes.** (**A**) Correlation analysis between SNRPB and oxidative stress-related pathways in MSigDB database. (**B**) Correlation analysis between SNRPB and oxidative stress-ferroptosis related genes in GeneCards and FerrDb databases. The color of sectors indicates the correlation (blue = positive, red = negative). The darker the sector color and the larger the sector area, the larger the correlation coefficient. The purple line means *p* < 0.01, the red line means *p* < 0.05, the green line means *p* < 0.001, and the line from thick to thin means the correlation between SNRPB and oxidative stress-ferroptosis related genes from large to small.

The SNRPB risk score was significantly correlated to 17 of 26 oxidative stress pathways from the MSigDB database (Figure 7A). SNRPB expression was significantly correlated to 68 of 88 oxidative stress-ferroptosis related genes expression from GeneCards and FerrDb database (Figure 7B). These results further support that the inhibition of SNRPB on HCC cell proliferation may be related to oxidative stress and ferroptosis.

### SNRPB knockdown combined with sorafenib inhibits tumor growth *in vivo*

**Figure 8:**
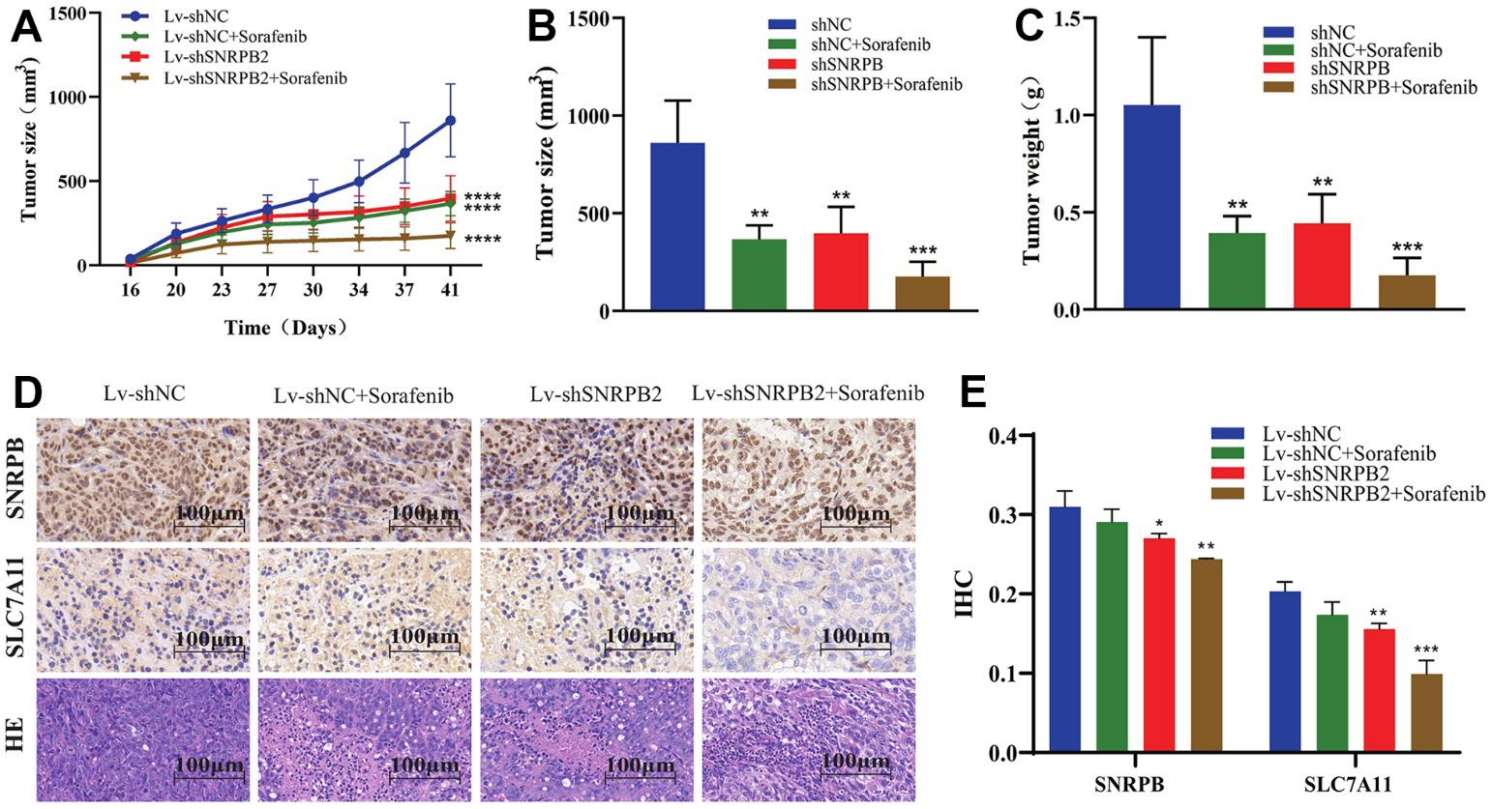
**SNRPB knockdown combined with sorafenib treatment inhibits tumor growth *in vivo*.** (**A**) Tumor volume curves differences of each nude mouse xenograft model (Lv-shNC, Lv-shNC+Sorafenib, Lv-shSNRPB2, and Lv-shSNRPB2+Sorafenib). (**B**, **C**) Tumor size and weight differences of each nude mouse xenograft model. (**D**, **E**) The IHC staining and HE staining analysis the expression levels of SNRPB and SLC7A11 in the xenograft tumors.

Our previous *in vitro* experiments found that the downregulation of SNRPB could cause the downregulation the ferroptosis-related gene, SLC7A11. Sorafenib induces the death of liver cancer cells via ferroptosis [14]. To further study the effect of SNRPB in combination with sorafenib on the growth of tumor cells *in vivo*, a nude mice xenograft tumor model was established through intraperitoneally injecting sorafenib when the tumor grew to 100 mm^3^. Results show that the tumor volume and weight of the Lv-shNC+Sorafenib, Lv-shSNRPB2, and Lv-shSNRPB2+Sorafenib groups were significantly reduced, compared to the control group, and the tumor volume and weight of Lv-shSNRPB2+Sorafenib group were the smallest (Figure 8A–8C). The Immunohistochemistry (IHC) staining results demonstrated that the SLC7A11 expression decreased significantly in the Lv-shNC+Sorafenib and the Lv-shSNRPB2 groups, and that SNRPB knockdown combined with sorafenib enhanced this effect (Figure 8D, 8E). These results indicate that SNRPB knockdown can be used in combination with sorafenib to increase its efficacy.

## DISCUSSION

In this study, we found that SNRPB had high diagnostic value in distinguishing HCC from paracancerous tissues, and the high expression of SNRPB was associated with poorer outcome of HCC patients. Peng et al. [9] conducted a research based on the association of C-MyC-mediated SNRPB upregulation and the occurrence/development of HCC, which is consistent with our results. Besides that, our study further confirmed that SNRPB was an independent prognostic factor of HCC as shown through Cox analyses. A nomogram and calibration curve were constructed to demonstrate the accuracy of using SNRPB expression to forecast the survival rates of HCC patients. Additionally, single cell sequencing analysis showed that HCC cell subset with high expression of SNRPB accounted for a higher proportion in HCC cells with higher stages.

Immune infiltration plays a vital role in tumorigenesis and tumor progression, which has an adverse effect on the outcome of tumor patients [15]. Our study demonstrated that SNRPB was significantly related to several key immune inhibitors and chemokines and was significantly negatively related to the immune stroma score, while SNRPB expression showed significant differences between different HCC immune subtypes. Single cell RNA sequencing analysis also demonstrated that SNRPB was expressed at high levels in cancer-associated immune cells. Therefore, this research indicates that SNRPB is concerned with HCC progression and affects the prognosis of HCC patients, while SNRPB may play a role in immunotherapy.

As previously reported, SNRPB can promote HCC cell proliferation [9], but it is unclear whether SNRPB has an impact on cell cycle. Through single cell sequencing analysis, we found that the expression of CDK1, CDK4, CCNB1, etc. [16, 17], which promote cell cycle progression were upregulated in HCC_SNRPB_High cell subset, and HCC_SNRPB_High cell subset was mainly related to cell cycle-related functions and pathways. Therefore, upregulation of SNRPB may promote HCC progression by affecting cell cycle-related genes and pathways. Through *in vitro* experiments we found that SNRPB knockdown induced G2/M arrest, and the number of replicating DNA molecules in the cells was significantly reduced, along with the expression levels of CyclinB1, CDK1, and CDK4. Together, our results indicate that SNRPB might act through the CDK1/CyclinB1 pathway to regulate HCC cell proliferation. It is well recognized that tumor development is linked to cell cycle [18–20], and the activation and deactivation of cyclin-dependent kinases (CDKs) can regulate cell cycle processes [15]. Inhibition of CDK1, the only CDK required for the cell cycle [21], can control G2/M transition and reverse DNA-damage sensitivity [22]. CyclinB1 (CCNB1) is a protein that activates specific CDKs required for cell cycle progression [23]. During karyomitosis, the activity of CDK1 is maintained by CyclinB1-CDK1 [22], CDK1, and the CDK1/CyclinB1 pathway [23]. CDK1 can also interact with Sox2 to increase the stemness of lung cancer cells [24]. ESRRA regulates the CD/CDK1/CyclinB1 pathway through DSN1 and promotes the development of gastric cancer [25].

Single cell sequencing analysis found that the expression levels of NOX4, NOXA1, NOXO1, etc., which promote oxidative stress [26] and NCOA4, TF and IDH1, which promote ferroptosis [27], were upregulated in HCC_SNRPB_Low cell subset. The expression levels of SOD3, CAT, GPX7, etc., which inhibit oxidative stress [26, 28], and GPX4, SLCA11 and RPL8, which inhibit ferroptosis, were downregulated in HCC_SNRPB_Low cell subset. These results indicate that downregulation of SNRPB may promote HCC cell death by inducing oxidative stress and ferroptosis. GO-KEGG analysis also indicated that SNRPB was linked to oxidative stress and ferroptosis. GSEA analysis showed that low SNRPB expression activates genes linked to oxidative stress and ferroptosis. SNRPB expression was significantly positively related to the KEAP1 [29] and TCPTP [30] expression which are closely related to oxidative stress in HCC, and SLC7A11, GPX4 and ACSL4 expression which are closely related to ferroptosis in HCC [31]. Moreover, our study showed that SNRPB was significantly related to oxidative stress-related pathways and oxidative stress-ferroptosis related genes. Therefore, it is possible that SNRPB is involved in oxidative stress and ferroptosis in HCC. Keap1-Nrf2-ARE pathway has been shown to be the most crucial pathway against oxidative stress [29]. Keap1 interacts with Nrf2 retaining it in the cytoplasm, which maintains Nrf2 at low levels by mediating its ubiquitination and degradation [32]. Deletion of Keap1 have disparate effects by different ferroptosis inducers in lung cancer cells [33]. Inhibition of Nrf2 significantly upregulates the expression of S1R in HCC cells induced by sorafenib, while inhibition of S1R improves the sensitivity of HCC cells to sorafenib [34].

Sorafenib is a multikinase inhibitor which has been approved by FDA to treat HCC [35]. HCC cell death is induced by sorafenib through ferroptosis [14]. It also has been identified as an inhibitor of SLC7A11 [36] by inhibiting the its activity and inducing ferroptosis *in vivo* [37, 38]. Numerous studies have shown that SLC7A11 has become a central hub between ferroptosis and tumor suppression [39–41]. We found that SLC7A11 expression was downregulated along with the downregulation of SNRPB expression in Huh7 and Hep3B cell lines. SNRPB knockdown combined with sorafenib treatment could significantly reduce tumor volume and weight in subcutaneous xenografted nude mice models. Besides, SLC7A11 expression in the tumor was also significantly decreased. These results indicate that SNRPB knockdown can promote ferroptosis induced by sorafenib and may be related to oxidative stress, and that SNRPB knockdown combined with sorafenib drug therapy can lead to better efficacy. Drug resistance is one of the most critical markers of cancer. Certain novel treatment strategies have focused on developing targeted therapies. For example, DDR2 can drive sorafenib resistance by the NF-κB/c-Rel signaling pathway in HCC [42], while ABCC5 promotes sorafenib resistance by inhibiting SLC7A11-induced ferroptosis in HCC [43]. The development of these targets provides novel methods of optimizing the application of sorafenib for tumors therapies. Our results suggest that SNRPB may have a great potential to overcome sorafenib resistance by affecting ferroptosis.

## CONCLUSIONS

In brief, SNRPB may be an independent prognostic factor related to HCC progression and may play a role in immunotherapy. Moreover, it provides the first evidence that SNRPB knockdown not only induces G2M phase arrest and restrain cell proliferation, but also downregulates SLC7A11 expression, which is closely associated to ferroptosis. Additionally, SNRPB knockdown can enhance anticancer activity induced by sorafenib, which may offer a novel target for HCC therapies. Furthermore, previous studies on HCC therapies have been limited to immunotherapy, cell cycle, oxidative stress or ferroptosis, our study explored the role of SNRPB in HCC therapies from the perspective of the immunotherapy, cell cycle, oxidative stress and ferroptosis combination. Results suggest that SNRPB is a therapeutic target that can be integrated into immunotherapy, cell cycle therapy and sorafenib-induced ferroptosis, which provides a novel basis for HCC therapies, although the immune mechanism of SNRPB in HCC cells needs further investigation in a clinical setting.

## MATERIALS AND METHODS

### Relationship between SNRPB and the prognosis of HCC

Downloaded transcriptional expression data and relevant clinical data of 374 liver hepatocellular carcinoma (LIHC) and 50 paired adjacent normal samples data from TCGA. We used ggplot2 package to analyze differences of SNRPB expression in 50 HCC and 50 paired adjacent normal samples. We used DESeq2 [44] to find out DEGs between SNRPB data sets with high and low expression (cut-off value of 50%), |log fold change (logFC)| > 1 and *p* < 0.05 since the difference was statistically significant.

We adopted pROC software package to conduct the receiver operating characteristic (ROC) analysis in 374 HCC as well as 50 paired adjacent normal tissues. The range of 0.5 to 1.0 calculated AUC indicated 50-100% discrimination power.

We adopted Cox regression analysis on the following variables: T, N, M stages, histologic grade, tumor status, and SNRPB, and used the ggplot2 package to construct forest plots. Then, used the RMS package and survival packages to construct nomogram and calibration curves.

We assessed the prognostic effect of SNRPB expression among HCC patients through Cox regression analyses, while SNRPB prognostic value was verified using a Kaplan-Meier Plotter.

We obtained transcriptional expression data and relevant clinical data of 212 LIHC samples data (ICGC database), and analyzed differences of SNRPB expression in 374 LIHC (TCGA database) and 212 LIHC (ICGC database) samples at different HCC stages using Wilcoxon test.

### Single cell RNA sequencing analysis of SNRPB

10 LIHC samples and 8 adjacent normal samples were acquired from GSE149614. Seurat package was used for further quality control, data normalization, dimension reduction and unsupervised clustering analysis of single cell datasets. Harmony package was used to normalize and standardize the single cell datasets. The RunPCA function was used to reduce the dimensionality of the datasets. The FindClusters function was used to find out the major cell clusters (resolution = 1.5). Visualization was performed using t-SNE.

We adopted clusterProfiler package to preform GO-KEGG enrichment analysis of DEGs between two cell subsets (HCC_SNRPB_High and HCC_SNRPB_Low).

### GSEA

We adopted clusterProfiler [45] to conduct a GSEA of SNRPB datasets with high and low expression levels. *p* < 0.05, false-discovery rate (FDR) < 0.25 and |NES| > 1 were considered statistically different.

### Relationship between SNRPB and genes related to cell cycle, oxidative stress and ferroptosis

We obtained 26 oxidative stress-related gene sets (MSigDB). Then, we obtained the SNRPB risk score of 26 gene sets by ssGSEA, and adopted Hmisc package to explore the correlation between the SNRPB risk score and the 26 gene sets. We adopted Corrplot package to draw the correlation heatmaps. The higher SNRPB risk score, the worse prognosis of HCC.

We obtained 8989 oxidative stress-related genes from GeneCards and 111 ferroptosis-related genes from FerrDb. We adopted Venn Diagram to take the intersection of these genes and obtained 88 genes related to oxidative stress and ferroptosis. We adopted Hmisc package to explore the correlation between the SNRPB and the 88 genes, then used Corrplot package to draw the correlation heat map.

### Immune infiltration

We analyzed the association of SNRPB and the level of immune infiltration, immune characteristics (immune checkpoints, immune inhibitors, and chemokines), immune matrix score and LIHC immune subtypes through TISIDB [46]. We adopted Estimate package to assess the association of SNRPB and immune stroma score.

### Specimens and cell lines

In total 30 paired HCC specimens and adjacent normal tissues were obtained from Affiliated Hospital of Guilin Medical University. The clinical features of the patients are summarized in Supplementary Table 1. Human HCC cell lines Huh7 and Hep3B were preserved and subcultured in our laboratory.

### Cell culture and transfection

Cells were grown at 37° C with 5% CO2 using DMEM (Gibco, C11965500BT) with 10% FBS (Gibco, A3160802) and 1% Penicillin/Streptomycin (Gibco, 15140122). SNRPB knockdown lentivirus (Lv-shSNRPB1/Lv-shSNRPB2) and the negative control (Lv-shNC) were purchased from GenePharma Co. Ltd. Cells were transfected and screened using puromycin (Sigma-Aldrich, P9620) for 4 days. Target sequences were listed below, Lv-shSNRPB1: ccACAAGGAAGAGGTACTGTT, Lv-shSNRPB2: ccTCCCAAAGATACTGGTATT, Lv-shNC: TTCTCCGAACGTGTCACGT.

### RT-qPCR

RT-qPCR was used to detect 30 paired HCC specimens and adjacent normal tissues. TRIzol (Invitrogen, USA) was used to extract the RNA, while HiScript Reverse Transcriptase (Vazyme, China) was used to construct cDNA. RT-qPCR was performed on a Quantstudio 6 (ABI, Singapore) system by SYBR Green Master Mix (Vazyme, China). PCR primer sequences: GAPDH, forward: 5′-TCAAGAAGGTGGTGAAGCAGG-3’, reverse: 5’-TCAAAGGTGGAGGAGTGGGT-3’; SNRPB, forward: 5’-TCGCTTCTCTTCCCTTTC-3’, reverse: 5’-TCTTCATTGGCACCTTCA-3’. The relative expression levels were expressed in terms of 2^-ΔCt^ (ΔCt is compared to the actin control).

### Western blotting

Total proteins were extracted from cells with RIPA lysate buffer (Beyotime, China). Western blot analysis was performed using anti-SNRPB (1:1000, 16807-1-AP, Proteintech Group, Inc), anti-GAPDH (1:1000, AB-P-R 001, Hangzhou Goodhere Biotechnology Co. Ltd), anti-SLC7A11 (1:1000, 26864-1-AP, Proteintech Group, Inc), anti-CDK1 (1:1000, 19532-1-AP, Proteintech Group, Inc), anti-CDK4 (1:2000, 11026-1-AP, Proteintech Group, Inc), as well as anti-CyclinB1 antibodies (1:2000, 55004-1-AP, Proteintech Group, Inc). After incubation with the secondary antibody (1:50000, Wuhan Boster Biological Technology, Ltd), which was conjugated with horseradish peroxidase (HRP). Chemiluminescent substrate was used to detect the protein bands (P1050, Applygen), which were exposed to X-ray films (Carestream Health, Inc). The ratio between the gray values of the target protein band and GAPDH band was used as the target protein expression level.

### Cell cycle detection

Lv-shSNRPB1/Lv-shSNRPB2 and Lv-shNC Cells were serum-starved for overnight to induce cell cycle synchronization and those in the logarithmic growth phase were collected for overnight immobilization in 70% ethanol at -20° C. Thereafter, washed and incubated with PI and analyzed using flow cytometry (CytoFLEX, Beckmancoulter).

### Cell proliferation assay

The proliferation abilities of the Lv-shNC, Lv-shSNRPB-1, and Lv-shSNRPB-2 transfected cells were detected by using a BeyoClickTM EDU-594 Kit (C0078S, Beyotime), and photographed by a fluorescence microscope (Msho, MF53). Cells were double stained using EdU (5-ethynyl-2’-deoxyuridine) emitting a red fluorescence to detect newly synthesized DNA, while using DAPI (4’,6-diamidino-2-phenylindole) to label the nuclei.

### Xenograft models

Randomly divided 4-week-old BALB/C nude mice (Sike Jingda Laboratory Animal Co. Ltd) into two groups, Lv-shNC and Lv-shSNRPB2 (n = 12/group). They were subcutaneously inoculated with 4×10^6^ Huh7 cells and were divided into 4 groups: Lv-shNC, Lv-shSNRPB2, Lv-shNC+Sorafenib, and Lv-shSNRPB2+Sorafenib. When the tumor grew to 100mm^3^, mice in the Lv-shNC+Sorafenib and Lv-shSNRPB2+Sorafenib groups were intraperitoneally injected with sorafenib (sorafenib was formulated using a sterile solution and the injection dose used was 10mg/kg [47]), while Lv-shNC and Lv-shSNRPB2 mice were injected with the sterile solution, every other day for 2 weeks. Tumor volume was continuously measured during the experiment. The mice were euthanized 25 days later using a 2% pentobarbital sodium injection and death was confirmed by cervical dislocation.

### IHC

Tissue sections were dewaxed using xylene, rehydrated with ethanol (100%-95%-80%), treated with H2O2, boiled at high pressure in sodium citrate buffer, blocked with 5% BSA, then incubated with anti-SNRPB and anti-SLC7A11 antibodies. Subsequently, they were incubated using the secondary antibody conjugated with HRP. They were followed by DAB staining. The IHC staining images were captured by light microscope, and the optical density index was determined using an optical densitometer.

### Hematoxylin-eosin staining (HE)

Tissue sections were decolorized with xylene and ethanol, and stained with hematoxylin to stain the nucleus. The excess hematoxylin was removed by washing in tap water, and then the cytoplasm was stained with eosin. Representative images of HE staining were captured using an optical microscope (Leica, DM750P).

### Statistical analysis

Graphs and statistical analyses of data obtained from TCGA, MSigDB, GeneCards and other databases were performed using R software (3.6.3) [48]. All experimental data was analyzed and displayed using GraphPad Prism 8. The comparison between two groups was calculated using Student’s T test, and for three or more groups, one-way ANOVA was used. (*p < 0.05, **p < 0.01, and ***p < 0.001).

### Availability of data and materials

The datasets used and analyzed during the current study are available from the corresponding author on reasonable request.

## Supplementary Material

Supplementary Table 1
